# Precision medicine requires precision proteomics: discordance between proteomic and clinical assays in UK Biobank

**DOI:** 10.1093/cvr/cvaf167

**Published:** 2025-09-26

**Authors:** Bhawana Singh, Ioanna Tzoulaki, Manuel Mayr

**Affiliations:** National Heart and Lung Institute, Imperial, 86 Wood Lane, London W12 0BZ, UK; Department of Epidemiology and Biostatistics, School of Public Health, Imperial College London, London, UK; Systems Biology, Biomedical Research Foundation of the Academy of Athens, Athens, Greece; National Heart and Lung Institute, Imperial, 86 Wood Lane, London W12 0BZ, UK; Division of Cardiology, Medical University of Vienna, Vienna, Austria

The UK Biobank (UKB) offers an unparalleled open access multi-omics resource, including 2923 plasma proteins measurements in 54 219 participants using the Olink Explore 3072 platform.^1^ It also provides protein quantitative trait locus (pQTL) data to support proteo-genomic analyses and causal inference.^1^ However, affinity-based proteomics platforms, such as Olink and SomaScan, can yield discordant results.^2,3^

Apolipoprotein B (ApoB) and lipoprotein(a) [Lp(a)] are known major risk factors for atherosclerotic cardiovascular diseases^4–6^. Yet, UKB studies using Olink-based apolipoprotein measurements and pQTLs have failed to confirm these associations.^7,8^ For example, Deng, Y.-T. *et al.*^7^ reported that apolipoprotein(a) [LPA]—the characteristic apolipoprotein of Lp(a)—was not significantly associated with the incidence of myocardial infarction (MI) after correcting for multiple testing [hazard ratio: 1.07 (1.00–1.15), nominal *P*-value: 0.035 Bonferroni-corrected threshold *P*-value < 1.7e^−5^, based on 2923 tests].

Even ApoB measured by Olink shows no association with 10-year incident major cardiovascular events in UKB, including MI, cardiac death, and cerebral infarction (*Figure 1A*). As previously reported, the Spearman correlation between Olink LPA and clinical assays for Lp(a) in UKB is high (*ρ* = 0.82) but poor (*ρ* = 0.08) for ApoB^1^ (*Figure 1B*). This highlights that genuine treatment targets can be overlooked when relying solely on affinity-based platforms. The Olink workflow requires dilutions of up to 1:100 000 to avoid a plateau effect for highly abundant plasma proteins. Yet, ApoB was measured at a 1:1000 dilution. Affinity-based proteomics platforms may also be affected by isoforms, post-translational modifications, or hydrophobicity-related losses, all contributing to low correlation.

**Figure 1:**
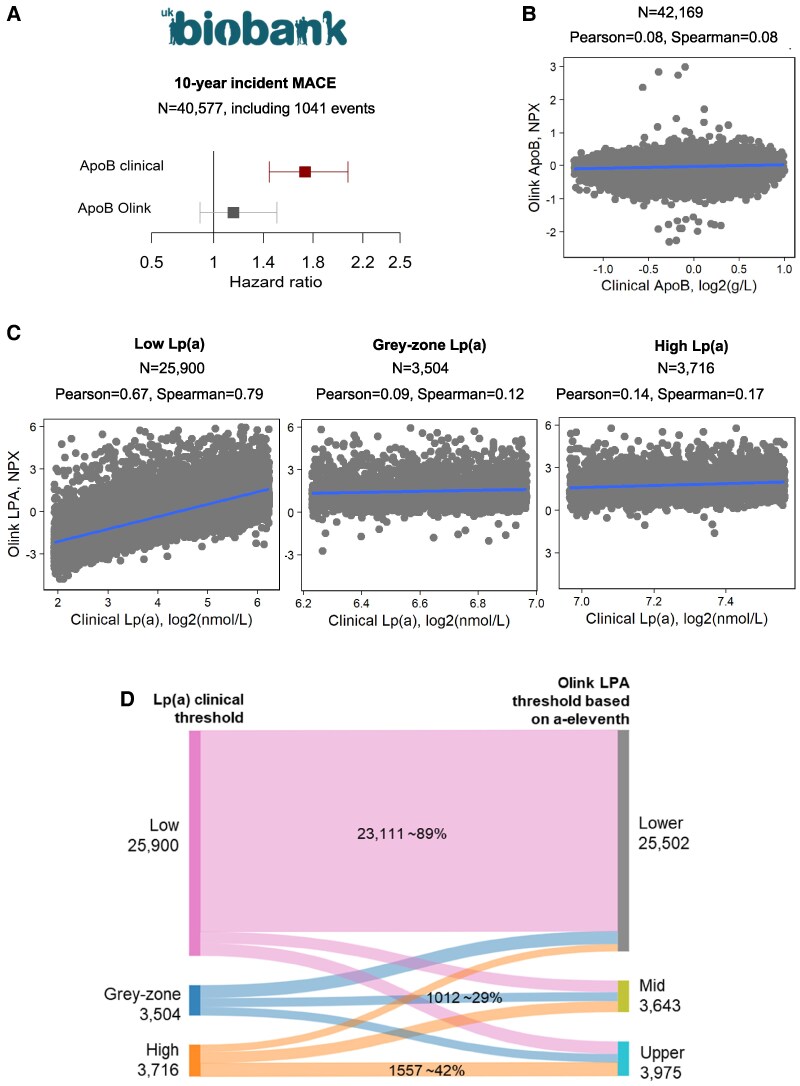
Key apolipoproteins for atherosclerotic cardiovascular disease in UK Biobank. (*A*) Forest plot shows age- and sex-adjusted hazard ratio (HR) with 95% confidence interval computed using Cox proportional hazards model. HR is plotted for 10-year incident major cardiovascular events (MACE) association with apolipoprotein B (ApoB) clinical and Olink assays in *N* = 40 577 participants. Clinical ApoB values were log2 transformed. Individuals with prior or at baseline atherosclerosis (ICD10: I70), or ischaemic heart diseases including myocardial infarction (ICD10: I20-I25) or cardiac arrest including sudden cardiac death (ICD10: I46) or cerebral infarction (ICD10: I63) were excluded from analysis. Grey indicates *P*-value >0.05, and maroon indicates *P*-value < 0.05. (*B*) Scatterplot illustrates the correlation between ApoB measured by Olink (*y*-axis) and clinical (*x*-axis) assays in *N* = 42 169 participants. The line in the scatterplot shows fitted linear regression. (*C*) Scatterplot displays the correlation between lipoprotein(a) [Lp(a)] clinical assay (*x*-axis) and apolipoprotein(a) [LPA] Olink assay (*y*-axis) across participants with low (<75 nmol/L, *N* = 25 900), grey-zone (≥75 nmol/L and ≤ 125 nmol/L, *N* = 3504), and high (>125 nmol/L, *N* = 3716) Lp(a). (*D*) Sankey diagram shows the reclassification of clinical Lp(a) threshold by Olink LPA based on a-eleventh quantile. The text in the flow highlights the percentage classified correctly by Olink LPA. Participants who had withdrawn or had missing values for Olink or clinical assays were excluded. Only baseline measurements were used for this analysis conducted under UKB research application 221022102. Statistical analysis and associated figures were generated with R version 4.2.2.

Although, the overall correlation between Olink LPA and clinical Lp(a) was high (*ρ* = 0.82),^1^ stratifying the UKB cohort by the clinical reference ranges revealed substantial variability. In UKB participants with low Lp(a) (<75 nmol/L, *N* = 25 900) the correlation remained strong (*ρ* = 0.79, *P*-value <2.2e^−16^). However, it dropped markedly in the grey-zone (≥75 nmol/L, ≤125 nmol/L, *N* = 3504) and the high (>125 nmol/L, *N* = 3716) Lp(a) groups, with correlations of 0.12 (*P*-value = 1.4e−12) and 0.17 (*P*-value <2.2e^−16^), respectively (*Figure 1C*). This Olink LPA example demonstrates that a high overall correlation does not preclude dynamic range limitations of the assay.

Based on the clinical threshold for Lp(a), 78% of participants (*N* = 25 900) had low levels (<75 nmol/L), ∼11% (*N* = 3716) high (>125 nmol/L), and ∼11% (*N* = 3504) fell into the grey-zone. To approximate these thresholds, Olink LPA measurements were divided into eleven quantile groups, each representing ∼11% of the data. Participants in the top 11% (highest values) were classified as ‘Upper’, the next 11% as ‘Mid’, and the remaining 78% as ‘Lower’. A Sankey diagram comparing clinical Lp(a) thresholds with quantile-based Olink LPA thresholds revealed substantial misclassification, particularly among higher-risk individuals (*Figure 1D*). Only 42% of participants with high Lp(a) and 29% in the grey-zone were correctly classified.

We highlight notable gaps in the Olink assay for quantifying apolipoproteins. Beyond the poor correlation for ApoB, the example of clinical Lp(a) vs. Olink LPA shows that discordance occur despite strong overall correlation. Individuals with low Lp(a) were reliably quantified, whereas higher-risk groups were underestimated. In these groups, Lp(a) concentrations exceeded the measurement range, and quantitation became imprecise due to a plateau effect.

While cis-pQTLs are often considered as evidence that an affinity-based proteomics assay detects the intended protein, this interpretation requires caution. Even a single amino acid substitution can alter epitopes and impair binding affinity. Unless binding affinity is demonstrated to be unaffected by genetic variants, attributing a pQTL to genuine variation in protein abundance variance remains uncertain. Besides epitope effects, cross-reactivity, limited sensitivity, and narrow dynamic range can all impair assay performance. Robust validation requires orthogonal methods—such as targeted mass spectrometry with reference peptides, validated immunoassays, and depletion and spike-in experiments—rather than reliance on genetic evidence alone. Replication in independent cohorts provides supportive evidence, but repeating measurements on the same affinity-based platform only reproduces its limitations.

The UKB continues to lead the way in adopting cutting-edge technologies to advance our understanding of human health and disease. Olink proteomics will be expanded to all participants, while SomaScan in 50 000 participants will enable cross-platform validation.^3^ Gaps in apolipoprotein quantification highlight the need to filter out less reliable assays.^9,10^ Looking ahead, incorporating targeted mass spectrometry could further improve the depth and precision of protein quantification. Heavy labelledproteotypic peptides, which differ in mass from endogenous analytes, would provide a reference method to assess the quality of affinity binders, thereby strengthening the translational potential of UKB proteomics data.

## Authors’ contributions

B.S. and M.M.: Study conception; B.S.: data analysis, interpretation, and draft of the article; M.M.: critical revision and final approval; I.T.: UKB resource and manuscript review; B.S. and M.M. had primary responsibility towards final content. All the authors have read and approved the final manuscript.

## Data Availability

Underlying clinical and Olink data are available through the UK Biobank Research Analysis Portal (https://www.ukbiobank.ac.uk/enable-your-research).
